# Metabolic, hemodynamic and structural adjustments to low intensity exercise training in a metabolic syndrome model

**DOI:** 10.1186/1475-2840-12-89

**Published:** 2013-06-18

**Authors:** Eduardo Morvan, Nathalia Edviges Alves Lima, Jacqueline Freire Machi, Cristiano Mostarda, Kátia De Angelis, Maria Cláudia Irigoyen, Rogério Brandão Wichi, Bruno Rodrigues, Laura Beatriz Mesiano Maifrino

**Affiliations:** 1 Human Movement Laboratory, Sao Judas Tadeu University, Av. Taquari, 546, São Paulo/SP 03166-000, Brazil; 2 Hypertension Unit, Heart Institute (InCor), Medical School of University of Sao Paulo, São Paulo/SP, Brazil; 3 Translational Physiology Laboratory, Nove de Julho University, São Paulo/SP, Brazil; 4 Federal University of Sergipe, Aracajú/SE, Brazil; 5 Institute of Cardiology Dante Pazzaneze, São Paulo/SP, Brazil

**Keywords:** Fructose, Exercise training, Triglycerides, Insulin resistance, Baroreflex sensitivity, Cardiovascular remodeling, Aorta remodeling

## Abstract

**Background:**

The increase in fructose consumption is paralleled by a higher incidence of metabolic syndrome, and consequently, cardiovascular disease mortality. We examined the effects of 8 weeks of low intensity exercise training (LET) on metabolic, hemodynamic, ventricular and vascular morphological changes induced by fructose drinking in male rats.

**Methods:**

Male Wistar rats were divided into (n = 8 each) control (C), sedentary fructose (F) and ET fructose (FT) groups. Fructose-drinking rats received D-fructose (100 g/l). FT rats were assigned to a treadmill training protocol at low intensity (30% of maximal running speed) during 1 h/day, 5 days/week for 8 weeks. Measurements of triglyceride concentrations, white adipose tissue (WAT) and glycemia were carried out together with insulin tolerance test to evaluate metabolic profile. Arterial pressure (AP) signals were directly recorded. Baroreflex sensitivity (BS) was evaluated by the tachycardic and bradycardic responses. Right atria, left ventricle (LV) and ascending aorta were prepared to morphoquantitative analysis.

**Results:**

LET reduced WAT (−37.7%), triglyceride levels (−33%), systolic AP (−6%), heart weight/body weight (−20.5%), LV (−36%) and aortic (−76%) collagen fibers, aortic intima-media thickness and circumferential wall tension in FT when compared to F rats. Additionally, FT group presented improve of BS, numerical density of atrial natriuretic peptide granules (+42%) and LV capillaries (+25%), as well as the number of elastic lamellae in aorta compared with F group.

**Conclusions:**

Our data suggest that LET, a widely recommended practice, seems to be particularly effective for preventing metabolic, hemodynamic and morphological disorders triggered by MS.

## Background

Metabolic syndrome (MS), according to International Diabetes Federation [1], is clinically characterized by central obesity, and at least two of these risk factors: high triglyceride levels; low HDL cholesterol; high blood pressure levels; and fasting plasma glucose levels increase. These risk factor associations generally are related with increased risk of diabetes [2] and cardiovascular mortality [3]. The link between insulin resistance, inflammation and obesity [4] is the most widely accepted hypothesis for the development of MS, and treatment should address each one of these elements. Furthermore, high dietary fructose intake has been found to contribute to increased prevalence of MS [5,6].

Clinical and epidemiological evidence suggest a progressive association between fructose consumption and the obesity epidemic along with other abnormalities which are seen in MS [7]. In this sense, fructose overload in drinking water or chow has been used to promote metabolic, hemodynamic, structural and functional derangements in rodents. This MS model has been used by our group in order to understand the various aspects of obesity, dyslipidemia and insulin resistance-associated cardiovascular changes [8-11]. In addition, fructose overload in rats has been linked to negative cardiac remodeling [9], as demonstrated by increased heart-to-body weight ratio, myocyte diameter, as well as left ventricular fibrosis and perivascular collagen type III deposition [12].

Aerobic exercise training (ET) may effectively mitigate fructose-induced hypertension in rats [13], and it has been acknowledged as a significant component in the treatment and prevention of human cardiovascular disease [14]. In fact, we has recently demonstrated that moderate intensity ET prevented diastolic dysfunction in fructose male rats [9], attenuated metabolic impairment, resting tachycardia, cardiac and vascular sympathetic increases, and baroreflex sensitivity decrease induced by fructose overload in ovariectomized female rats [10]. Additionally, walking also correlated positively with metabolic and hemodynamic changes in fructose male rats [11]. However, the effects of walking, a low intensity ET (LET), on metabolic and hemodynamic parameters induced by fructose overload and its consequences on the cardiac and aortic remodeling remains poorly understood. Thus, the aim of this study was to evaluate the effects of 8 weeks of LET on metabolic, hemodynamic, as well as on cardiovascular and arterial morphological changes induced by fructose drinking in male rats. To our knowledge, this is the first study that addresses this issue.

## Methods

Experiments were performed in male Wistar rats (100 – 120 g, ~30 days old) from the Animal House of the São Judas Tadeu University, São Paulo, Brazil. We chose male rats due to the high prevalence of MS and the elevated incidence of cardiovascular mortality in men [3]. The animals were housed in collective polycarbonate cages in a temperature-controlled room (22°C) with a 12 h dark–light cycle (light 07:00 – 19:00 h). Rats were fed standard laboratory chow. The experimental protocol was approved by the Ethical Committee in Research of the Sao Judas Tadeu University (CEP - Protocol: 063/2006), and this investigation was conducted in accordance with the Principles of Laboratory Animal Care formulated by the National Institutes of Health (National Institutes of Health Publication No., 96–23, Revised 1996). The rats were randomly assigned into three groups: control (C, n = 8), sedentary fructose (F, n = 8), low intensity aerobic ET fructose (FT, n = 8).

### Fructose drinking and exercise training

Fructose overload was performed via dilution of D-fructose in the drinking water (100 g/L) for a total period of 18 weeks. Control animals received only water during this period [9,11]. Fructose consumption was measured daily, through the subtraction of the total volume given minus the remaining volume. The consumptions of chow and water (with or without fructose) were measured weekly. The total caloric intake was calculated considering that 2.89 kcal could be obtained from each consumed chow gram and that 4.0 kcal could be obtained from each ingested fructose gram [11].

During the tenth week of fructose overloading or water consumption, experimental groups were adapted to the treadmill (10 min/day; 0.3 km/h) and were submitted to a maximal treadmill exercise test. This exercise test was performed to determine aerobic capacity and ET intensity at the beginning of the exercise protocol (initial evaluation), after 4 weeks (to training intensity adjustments, data not show), and after ET protocol (final evaluation and aerobic capacity evaluation). Our group previously demonstrated that maximal treadmill exercise test can detect differences in aerobic performance, since that the maximum speed achieved in the test presented a good correlation with the maximum oxygen consumption [15]. In the eleventh week of fructose overload, ET was started and performed on a motorized treadmill at low intensity (20-30% of maximal running speed) to FT group, for 1 hour a day, 5 days a week for 8 weeks [9,11].

### Metabolic evaluations

At the initial (initial evaluation) and after 8 weeks of ET protocol (final evaluation) the blood glucose and triglyceride concentrations were measured using a Roche device (Accutrend GCT, Roche, São Paulo, Brazil) after four hours of fasting. For the intravenous insulin tolerance test (ITT), the rats were fasted for two hours and then anesthetized with thiopental (40 mg/kg body weight, i.p.). A drop of blood was collected from the tail to measure the blood glucose concentration using the Accucheck system (Roche, São Paulo, Brazil) before and 4, 8, 12, and 16 minutes after insulin injection (0.75 U/kg, i.p.), as previously described by Bonora et al. [16] and published by our group [9,11,17]. The constant rate of decrease of the blood glucose concentration (Kitt) was calculated using the 0.693/t1/2 formula. The t1/2 for blood glucose was calculated from the slope of the least squares analysis of the blood glucose concentrations during the linear phase of decline [9,11,16,17].

### Hemodynamic measurements

One day after final metabolic measurements, one catheter filled with 0.06 ml of saline were implanted in anesthetized rats (ketamine 80 mg/kg + xylazine 12 mg/kg, i.p.) into the carotid artery for direct measurements of the arterial pressure (AP), and into the jugular vein to vasoactive drugs administration (phenylephrine and sodium nitroprusside). One day after the catheter placement, the rats were conscious and allowed to move freely during the experiments. The arterial cannula was connected to a strain-gauge transducer (Blood Pressure XDCR, Kent© Scientific, Litchfield, CT, USA), and AP signals were recorded over a 30-min period by a microcomputer equipped with an analog-to-digital converter board (CODAS, 2-kHz sampling frequency, Dataq Instruments, Inc., Akron, OH, USA). The recorded data were analyzed on a beat-to-beat basis to quantify the changes in the mean AP (MAP) and the heart rate (HR) [9,11,17].

Sequential bolus injections (0.1 mL) of increasing doses of phenylephrine (0.25 - 32 mg/kg, i.v.) and sodium nitroprusside (0.05 - 1.6 mg/kg, i.v.) were given to induce increases or decreases in MAP responses (for each drug), ranging from 5 to 40 mm Hg. Baroreflex sensitivity was expressed as bradycardic response (BR) and tachycardic response (TR) in beats per minute per millimeter of mercury, as described elsewhere [10].

### Systolic arterial pressure variability

Systolic arterial pressure (SAP, systograms) was obtained from blood pressure records. Fluctuations in SAP were further assessed in the frequency domain by means of autoregressive spectral estimation. The theoretical and analytical procedures for autoregressive modeling of oscillatory components have been described previously [18,19]. Briefly, the SAP series derived from each recording were divided into 300 beat segments with a 50% overlap. The spectra of each segment were calculated via the Levinson-Durbin recursion and the order of the model chosen according to Akaike’s criterion, with the oscillatory components quantified in LF (0.2–0.6 Hz) and high-frequency (HF; 0.6–3.0 Hz) ranges [18,19].

### Tissue sample preparation

During the period of metabolic and hemodynamic evaluations (~1 week) the animals remained with fructose overload. Two days after hemodynamic measurements, the rats were killed. The animals were heparinized prior to fixation to optimize perfusion-fixation. White adipose tissue from different anatomical locations (perirenal, epididymal, mesenteric and subcutaneous) and the hearts (in diastole) were removed and weighed. The weight values of white adipose tissue presented in this study are the sum of the values taken from different anatomical locations. The myocardium was perfused through the aorta at a constant pressure of 80 mmHg using 0.1 M cacodylate buffer (3 min) followed by 2.5% glutaraldehyde solution diluted in cacodilate buffer. Posteriorly, the atria were separated from the ventricles, and the right from de left ventricle (LV) at the level of the papillary muscles, including the septum, was isolated. The ascending of aorta also excised and fixed with the same fixative solution for 24 h at temperature room.

### Right atria and section left ventricle

Fragments of the right atria and section that included the entire thickness of the LV wall were divided into slices of approximately 3 mm wide and 5 mm long, fixed in 2% paraformadehyde, 2.5% glutaraldehyde in 0.1 M cacodylate buffer for 2 h at 4°C and post-fixed in 1% osmium tetroxide in the same buffer for 2 h at 4°C. The fragments were dehydrated through a graded series of ethanol and embedded in Araldite. Thin sections were double-stained with uranyl acetate and lead citrate and examined with a JEOL – transmission electron microscope.

Fragments of the LV wall were fixed in formaldehyde solution 10%, buffered (pH 7.2) for 48 h, embedded in paraffin, and used for the histological slices, 6 μm thick, which were analyzed through polarized light microscopy with the use of Picrosirius staining in order to study the interstitial myocardial collagen fibers.

### Ultrastructural morphometry and stereology

Ten electron micrographs from right atria per animal, chosen by systematic random sampling of squares, were taken at a final magnification of ×15 000 and the numerical density of granules/field, volume density of ANP-granules, mitochondrial and myofibrils and the diameter of all granules present in the field were determined, using a computerized program (Axio Vision, Zeiss). For the volume density the electronmicrographs were analyzed by a stereological test-system with 82 points, and values were expressed as a percentage.

Two randomly chosen blocks from each LV, in which the myocytes were cut in cross section, were used for quantitative analysis. The ultra-thin sections were placed on a copper grid, and 10 randomly chosen fields per block were selected for micrographs, which were taken from specimens using the Jeol transmission electron microscope. Low power (x600) electron micrographs were used for quantitative analysis of the LV muscle tissue composition. Each electron micrograph was analyzed by the computerized program (Axio Vision, Zeiss) totalling 300 micrographs. The myocyte mean cross-sectional area (A[my]) was determined for every animal in each group. A test-system with 140 sampling points was put upon the monitor screen and calibrated.

The myocardium was analyzed considering the myocytes (my), capillaries (cap) and connective tissue (ct). The numerical density (Nv) of cardiomyocytes (my per mm3) and capillaries (cap per mm3) was determined [20]. The volume density was estimated for the myocyte (Vv [my]), capillaries (Vv[cap]) and collagens fibers (Vv[cf]): (*Vv*[*structure*] = *PP*[*structure*]/*PT*), where PP is the number of points that hit the structure, and PT is the total test-points. With the aid of the same test-system, the histological sections were used to estimate the volume density of the Picrosirius-stained collagen fibers was determined.

### Ascending aorta

The ascending aorta was dissected (from heart base to the aorta arch), removed and post-fixed in 4% paraformaldehyde in 0.1 mol/l phosphate buffer, pH 7.2 for 24 h. Aortic rings were dehydrated in graded ethanol concentrations (70, 80, 90 and 100%) and embedded in histological paraffin. The blocks were cut with a microtome (5 – μm – thick sections, Leica). Transverse sections were mounted on a glass slide and stained with the Haematoxylin-Eosin, Verhoff-Van Gienson e Picrosirius technique. Four slides with 5 semi-serial (1 section every 25 μm) sections each, i.e. a total of 20 sections were obtained from each sample. Morphological analysis conducted in a transversal aortic sections with a light microscope (Zeiss, x200 and x400 magnifications) permitted the identification of elastic lamellas (Verhoff-Van Gienson stain), smooth muscle constituents (Haematoxylin stain) and collagen fibers (Picrosirius stain).

### Morphological/morphometric analysis

Ascendant aorta images were acquired and digitized for off-line morphometric analysis (Image Pro Plus 5.1 software). Four measures per image were obtained at 0, 90, 180 and 270° to estimate intima and media thickness (IMT). The aorta mean cross-sectional area (A[my]) was determined for every animal in each group. The lumen area (a) was estimated by drawing a line over the circle delimited by the intima layer inner interface. The lumen diameter (d) was calculated as: *d* = 2 √ *a* /*π*. The mean cross-sectional area of the intima plus media (IMA, intima-media area) was estimated as: *IMA* = [*π*(*d*/2 + *IMT*)2 − *π*(*d*/2)2]. IMA data were corrected for tissue shrinkage due to fixation and further processing by multiplying by 1.28 (previously determined in a pilot study).

### Circumferential wall tension

Circumferential wall tension (CWT) was calculated by Laplace’s law as: *CWT* = *MSAP* × (*d*/2), where CWT is expressed in dyne/cm, MSAP is the mean of systolic arterial pressure (dynes/cm^2^), and d is the lumen diameter (mm) [21].

### Stereological analysis

Images were captured with a light microscope (Zeiss, x400 magnifications), and transferred to the image analysis program (Axio Vision Software, Zeiss). For volume density of the Picrosirius-stained collagen fibers, the photomicrographs of the aorta were analyzed by a stereological test-system with 200 points, and values were expressed as a percentage.

### Statistical analysis

Data are reported as mean ± SEM. After confirming that all continuous variables were normally distributed using the Kolmogorov-Smirnov test, statistical differences between the groups were obtained by 1-way analysis of variance (ANOVA) followed by the Student-Newman-Keuls post-test. Statistical differences between the data measured over time were assessed using repeated measures ANOVA. Pearson’s correlation was used to study the association between different parameters. All tests were 2-sided, and the significance level was established at P < 0.05. Statistical calculations were performed using SPSS version 17.0.

## Results

### Functional and metabolic parameters

The fructose groups presented higher daily liquid ingestion (F: 39.1±1.4 and FT: 38.7±1.0 ml/rat) and reduced daily chow consumption (F: 8.2±0.4 and FT: 9.1±0.3 g/rat) when compared to the C group (17.7±0.4 ml/rat and 13.1±0.2 g/rat, respectively) during experimental protocol. However, total caloric intake (kcal of fructose + kcal of chow) was similar among the studied groups (C: 30.8±1.2; F: 34.7±1.7; FT: 37.9±2.8 kcal/day).

Body weight was similar among all studied groups at the beginning of the protocol (~109±4 g). At the end of the study, body weight was similar between the C (440±9 g), F (438±10 g) and FT group (446±11 g) groups. The F group presented an increase in the visceral white adipose tissue weight (6.1±0.2 g) when compared to the C group (3.2±0.1 g), while LET was able prevent this increase, as observed in the FT group (3.1±0.2 g).

As shown in Table 1, there was no difference in exercise capacity between the experimental groups before and after LET protocol. At the beginning of the protocol, glycemic and triglyceride values were similar among studied groups. However, after LET or following protocols, glycemia remained unchanged in the experimental animals, while F and FT groups showed increased triglyceride levels when compared to C. Furthermore, it should be stressed that these values were reduced in the FT group when compared to the F group (Table 1). In the intravenous insulin tolerance test, the rate constant for blood glucose disappearance (KITT) was reduced in F and FT groups when compared to C. Nevertheless, LET protocol prevented the accentuated reduction of this parameter, as observed in FT group when compared to the F group (Table 1).

**Table 1:** Maximal running speed (MRS) and metabolic parameters before and after exercise training or following protocols in control (C), fructose (F), fructose low intensity trained (FT) groups

**Parameter / Group**	**C**	**F**	**FT**
**MRS (Km/h)**			
Before	1.11±0.11	1.21±0.07	1.13±0.06
After	1.34±0.07	1.25±0.08	1.54±0.11
**Glycemia (mg/dL)**			
Before	94±4	87±6	98±3
After	84±5	76±4	88±3
**Triglycerides (mg/dL)**			
Before	103±2	97±3	95±3
After	96±4	240±6*	160±10*†
**Kitt (%/min)**			
Before	4.0±0.2	5.0±0.3	5.4±0.6
After	5.0±0.3	2.8±0.3*	3.5±0.1*†

Values are expressed as mean ± SEM. Constant rate of decrease of the blood glucose concentration (Kitt). * p<0.05 vs. C; † p<0.05 vs. F.

### Hemodynamic measurements and systolic arterial pressure variability

Hemodynamic evaluations are presented in Table 2. Chronic fructose consumption increased systolic, diastolic and mean arterial pressure in F rats as compared with that in C rats. LET protocol was able to prevent the increase of systolic and mean arterial pressures in FT in comparison with F group. Baroreflex sensitivity, as evaluated by TR and BR triggered by AP rises and falls, was impaired in the F animals in comparison with C animals. However, LET prevented the TR and BR reduction, as observed in the FT group (Table 2). Similarly, LET prevented the increase of systolic arterial pressure variance (SAP Var) and LF band in FT animals in comparison with F (Table 2).

**Table 2:** Hemodynamic measurements and systolic arterial pressure variability in control (C), fructose (F), fructose low intensity trained (FT) groups

**Parameters /Group**	**C**	**F**	**FT**
**SAP (mmHg)**	122±2	151±3*	142±2*†
**DAP (mmHg)**	95±2	118±3*	112±4*
**MAP (mmHg)**	111±3	140±6*	125±5*†
**HR (bpm)**	325±12	344±10	352±4
**TR (bpm/mmHg)**	3.23±0.42	1.91±0.12*	2.55±0.11
**BR (bpm/mmHg)**	−1.71±0.12	−1.32±0.11*	−1.41±0.08
**SAP Var (mmHg**^**2**^**)**	23.5±2.6	41.9±6.5*	21.7±4.4†
**LF (mmHg**^**2**^**)**	3.0±0.5	8.1±0.6*	3.4±0.6†

Values are expressed as mean ± SEM. Systolic arterial pressure (SAP); Diastolic arterial pressure (DAP); Mean arterial pressure (MAP); Heart rate (HR); Tachycardic response (TR); Bradycardic response (BR); Systolic arterial pressure variance (SAP Var); Low frequency band of systolic arterial pressure variability (LF). * p<0.05 vs. C; † p<0.05 vs. F.

### Morphoquantitative study of the heart

**Figure 1:**
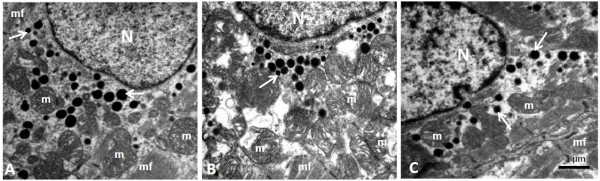
**Electron micrograph of right atrium cardiomyocyte of the C (panel A), F (panel B), and FT (panel C) groups.** The ANP-granules (arrows) are found at the pole of the nucleus (N), among numerous mitochondria (m), myofibrils (mf). Bar - 1 μm.

Table 3 shows the morphoquantitative data of atrial natriuretic peptide (ANP) granules in the right atria from experimental groups. ET prevented the reduction of numerical density of ANP granules, mitochondrial volumetric density, and increase of ANP granules area in FT group as compared with that in F group. Furthermore, FT animals increased mitochondrial volumetric density of the right atrium when compared to C and F animals (Figure 1).

**Table 3:** Secretory apparatus of atrial natriuretic peptide (ANP) in control (C), fructose (F), fructose low intensity trained (FT) groups

**Parameters /Group**	**C**	**F**	**FT**
**Nv[gr] /mm**^**3**^	22.1±2.3	10.8±1.4*	18.7±1.5†
**Vv[gr] (%)**	5.3±0.6	3.7±0.6	4.8±0.5
**Área (nm**^**2**^**)**	1785±40	2030±66*	1754±35†
**Vv[mit] (%)**	25.8±2.1	14.1±1.2*	32.7±1.9*†
**Vv[miofib] (%)**	23.9±2.1	23.1±1.9	23.7±2.3

Values are expressed as mean ± SEM. Numeric densities ANP granules (Nv[gr]); Volumetric density of granules (Vv[gr]), mitochondria (Vv[mit]) and myofibrils (Vv[miofib]). * p<0.05 vs. C; † p<0.05 vs. F.

**Figure 2:**
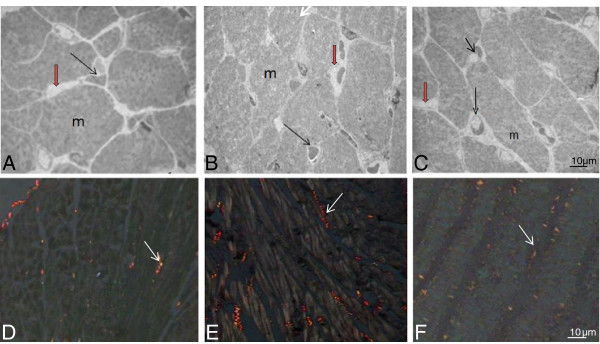
**Left ventricle from the C (panels A and D), F (panels B and E), and FT (panels C and F) groups.** Observe the interstitial (red arrow), collagen fibers (white arrows), capillaries (black arrows), and myocytes (m). Capillaries profiles are easily identified and muscle cell boundaries can be definitely visualized. Ultrathin sections of Epon-embedded tissue blocks **(A, B** and **C)** and Picrosirius stain **(D, E** and **F)**. Bar - 10 μm.

Morphoquantitative alterations of the LV triggered by fructose consumption and the effects of the LET are shown in Table 4 and Figure 2. Heart weight, heart weight / body weight ratio, area, numerical and volumetric densities of myocytes, as well as volumetric density of collagen fibers were increased in F animals when compared to C. Furthermore, numerical density of capillaries was reduced in the F group in comparison with the C group. Interestingly, LET prevented the impairment of these parameters, as observed in the FT group.

**Table 4:** Left ventricle morphoquantitative evaluations in control (C), fructose (F), fructose low intensity trained (FT) groups

**Parameters /Group**	**C**	**F**	**FT**
**HtW (g)**	1.31±0.04	1.42±0.01*	1.25±0.03†
**HtW / BW x 10**^**-3**^	2.8±0.1	3.4±0.1*	2.7±0.1†
**A[my] (μm**^**2**^**)**	479±18	588±22*	674±48*
**Nv[my] / mm**^**2**^	6.3±0.3	9.7±0.3*	8.0±0.5*†
**Nv[cap] / mm**^**2**^	5.4±0.3	3.9±0.1*	5.2±0.2†
**Vv[cf] (%)**	7.7±0.8	13.7±1.5*	8.7±1.5†
**Vv[my] (%)**	73.2±1.8	81.4±1.2*	74.8±1.6†
**Vv[cap] (%)**	3.6±0.5	3.1±0.3	3.6±0.4

Values are expressed as mean ± SEM. Heart weight (HtW); Heart weight body weight ratio (HtW/BW); Area of myocytes (A[my]); Numeric densities of myocytes (Nv[my]) and capillaries (Nv[cap]); Volumetric densities of collagen fibers (Vv[cf]), myocytes (Vv[my]) and capillaries (Vv[cap]). * p<0.05 vs. C; † p<0.05 vs. F.

### Morphoquantitative study of the aorta

**Figure 3:**
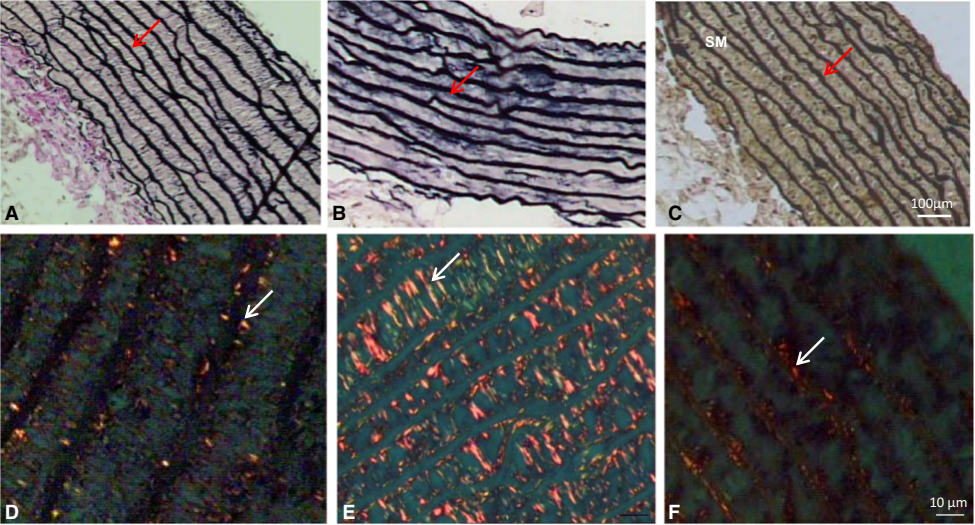
**Ascendant aorta from the C (panels A and D), F (panels B and E), and FT (panels C and F) groups.** Observe the elastic lamellas (red arrows) and collagen fibers (white arrows). Verhoff-Van Gienson stain **(A, B** and **C)** and Picrosirius stain **(D, E** and **F)**. Bar - 10 μm.

Morphoquantitative changes in the aorta are presented in Table 5. Chronic fructose overload induced important changes in the aorta artery of the F group, such as increase in area, diameter, intima-media thickness, volumetric density of collagen fibers, and circumferential wall tension, together with a decrease in the number of elastic lamellae (Figure 3) when compared to the C group. It is important to highlight that LET was able to prevent these morphological changes, as can be seen in the FT group.

**Table 5:** Aorta morphoquantitative evaluations in control (C), fructose (F), fructose low intensity trained (FT) groups

**Parameters /Group**	**C**	**F**	**FT**
**Area (mm)**	0.71±0.04	0.96±0.02*	0.65±0.01†
**Diameter (mm)**	0.95±0.02	1.15±0.05*	0.86±0.04†
**IM thickness (μm)**	158.1±0.7	177.3±3.1*	153.4±1.1†
**Vv[cf] (%)**	1.51±0.22	7.92±0.61*	1.92±0.04†
**EL number**	13.3±0.1	10.4±0.3*	12.5±0.2*†
**CWT**	62±3	81±5*	61±5†

Values are expressed as mean ± SEM. Intima media thickness (IM thickness); Volumetric density of collagen fibers (Vv[cf]); elastic lamellae numbers (EL numbers); circumferential wall tension (CWT). * p<0.05 vs. C; † p<0.05 vs. F.

### Correlations

Positive correlations were observed between white adipose tissue weight and heart weight / body weight ratio (r = 0.86; P < 0.0001), and with LV volumetric density of collagen fibers (r = 0.89; P < 0.0001), demonstrating that the increase in fat tissue may be associated with cardiac remodeling. As to hemodynamic data, a positive correlation was observed between systolic arterial pressure and intima-media thickness (r = 0.62; P = 0.0074). In addition, baroreflex sensitivity, as evaluated by tachycardic (r = 0.79; P = 0.0004) and bradycardic (r = −0.77; P = 0.0007) responses, were correlated with the number of elastic lamellae in all experimental animals, thus suggesting that aorta distensibility may be associated with responsiveness to pressure changes triggering a better baroreflex signaling*.*

## Discussion

The main finding of the present study lies in the fact that LET was able to prevent metabolic disorders, increase of arterial pressure values and variability, as well as baroreflex sensitivity impairment and peripheral sympathetic modulation in a MS model induced by high-fructose drinking. In addition, LET prevented cardiac and arterial structural changes, thus reducing collagen deposition, increasing the capillarity and the number of elastic lamellae, and increasing the number of atrial natriuretic peptide granules in the trained group.

It has been shown that fructose-fed rats present moderate hypertension and glucose intolerance, associated with high levels of plasma insulin, cholesterol and triglycerides [11,22-24]. In the present investigation, chronic fructose consumption induced an increase in triglyceride levels and arterial pressure, together with a decrease in insulin sensitivity, thus corroborating previous findings of our group [9,11].

Candidates underlying mechanisms for increments in the arterial pressure values and insulin resistance has been associated with increased sympathetic activity [25], and endothelial dysfunction due to reduction of endothelial nitric oxide synthase, and impairment of insulin receptor substrate 1 signaling pathways in muscle, liver, heart, kidney, and aorta [26,27]. Furthermore, uric acid production, hypertriglyceridemia, aldehyde formation, altered vascular reactivity, oxidative stress, augmented activity of renin angiotensin system, increased sodium reabsorption, and reduced baroreflex sensitivity have also been implicated [9,11,12,23,28,29]. In the present investigation, we observed a decrease in baroreflex sensitivity, as well as an increase of peripheral sympathetic modulation in the F group when compared to the C group. Thus, it is possible that the increase of arterial pressure has been influenced by autonomic derangements in the fructose rats.

ET was designed to reduce total cholesterol, triglycerides, systolic blood pressure, overweight/obesity, and diabetes mellitus, and has had a profound favorable impact on decreasing the incidence of initial and recurrent cardiovascular events [30,31]. In the present study, LET prevented the increase of circulating triglycerides and insulin resistance, reinforcing the role of physical training on lipid control and storage, as well as on the insulin sensitivity. It should be noted that the reduced adipose tissue observed in FT rats may be associated with these triglyceride and insulin resistance improvements. In this aspect, previous studies using ET as a therapeutic approach have described improvements on triglyceride and insulin profiles in fructose overload rats [9,11,32,33], reduction of inflammatory and oxidant status in type II diabetes [34-36], and Obese Zucker rats [37].

Regarding arterial pressure, although the FT group presented a slight but significant decrease in systolic blood pressure, the values were still higher when compared to C rats, revealing that LET was not able to totally prevent changes in this parameter. These findings corroborate a previous study carried out by our group [11], achieving lower blood pressure may be intensity-training dependent. In fact, Tjønna et al. [38] have demonstrated that high intensity aerobic interval training was more effective in promoting metabolic, physical capacity, and blood pressure improvements than continuous moderate exercise in MS individuals. The blood pressure improvements by high intensity ET observed by the authors were associated with greater benefit in the endothelial function of MS patients. They hypothesize that the low-and high-intensity ET programs differently affect shear stress on the walls of blood vessels during ET, and this yields that differences in molecular responses [38].

In the present study, the slight decrease of systolic arterial pressure after ET may be associated with the normalization of baroreflex sensitivity, reduction of systolic arterial pressure variability and peripheral sympathetic modulation in FT rats when compared to F rats. In this sense, our group has previously demonstrated that ET leads to changes in blood pressure mediated by baroreflex improvements in hypertensive [39], diabetic [40], dyslipidemic [41], and MS [9] animals. Thus, although we observed important autonomic benefits by low intensity ET, it is possible that endothelial function has not been fully restored, since it appears to be intensity training dependent [38], justifying the non-normalization of blood pressure in this study.

Our results demonstrated that, in addition to the metabolic and hemodynamic alterations promoted by high fructose drinking, the animals of F group displayed an increase in heart weight / body weight ratio, area, volumetric and numerical density of myocite, volumetric density of collagen fibers, together with a decrease in the numerical density of capillaries and ANP granules. Regarding ANP, previous evidence has shown that LV hypertrophy is favored by low levels of ANP, independently from blood pressure in hypertension [42]. More recently, Rubattu et al. [43] demonstrates that levels of N-terminal-proatrial natriuretic peptide are significantly reduced in hypertensive patients affected by MS, and they are significantly inversely related to the increased LV hypertrophy observed in these patients.

Similarly, arterial remodeling was also observed in F rats, since that these animals presented an increase in area, diameter, intima media thickness and circumferential wall tension, along with a reduction in the number of elastic lamellae. We may hypothesize that the increase in systolic arterial pressure together with increased aorta circumferential wall tension would account, at least in part, for arterial remodeling.

In addition, the loss of distensibility of the aorta and carotid arteries has been associated with changes in baroreflex sensitivity [44,45], since the mechanical stress of the arteries wall would not be able to adequately trigger the mechanoreceptors. Thus, the increase in volumetric density of collagen in the aorta of the F rats, as well as the correlations observed between the numbers of elastic lamellae with baroreflex sensitivity, seem to lend support to our contention that structural changes are associated with reduced autonomic function observed in the MS rats. Furthermore, we observed positive correlations between white adipose tissue and heart weight/body weight ratio, as well as with LV volumetric density of collagen fibers, suggesting that fat mass increments, and putative increased metabolic activity, may be associated with cardiac remodeling.

Few data exist with respect to how ET superimposed on hypertension impacts on LV and aorta remodeling. In the present study, LET was able to prevent the ventricular and aortic remodeling, normalizing heart weigh / body weight ratio, numerical density of capillaries, volumetric density of myocytes and collagen fibers in the LV, as well as improving intima-media thickness, volumetric density of collagen fibers, and circumferential wall tension in the aorta. Thus, it is possible that the changes induced by ET, as decrease of arterial pressure levels and arterial pressure variability, improvement of baroreflex sensitivity, as well as reduction of peripheral sympathetic modulation, have prevented ventricular and arterial pathological remodeling in trained animals. Furthermore, we cannot rule out the hypothesis that the normalization of numerical density of ANP granules may suggest peptide levels normalization, a fact that could also be associated with improved ventricular remodeling [43] in the trained group. However, future studies are necessary to better understand the possible mechanisms associated with this structural improvement.

This study has its limitations and opens opportunities for future research. First, although in this study comparative data with trained control group are not presented, we provided a range of in vivo and histological evidences about the effects of low intensity ET in a MS model. Moreover, the main focus of this study was to evaluate the effects of low intensity ET on the MS cardiovascular complications. Analysis of ANP activity, insulin concentration and receptors expression, as well as possible molecular mechanisms associated with the prevention of hemodynamic, autonomic and structural impairment by ET, were not performed in this study. Further studies are needed that address such issues.

## Conclusions

In conclusion, our data suggest that LET, a widely recommended practice, is particularly effective for treating the metabolic, functional and morphological disorders triggered by MS. In fact, although a normalization of systolic arterial pressure was not detected, improvements on insulin and baroreflex sensitivity, myocardial capillarity, aortic elastic lamellae, and circumferential wall tension, together with a reduction in white adipose tissue, collagen fibers in the aorta and LV, seem to offer strong support to the far-reaching beneficial effects of LET in the MS.

## Abbreviations

MS: Metabolic syndrome; ET: Exercise training; LET: Low intensity exercise training; ANP: atrial natriuretic peptide; C: Control group; F: Fructose group; FT: Fructose low intensity trained group; ITT: Insulin tolerance test; Kitt: Constant rate of decrease of the blood glucose concentration; SAP: Systolic arterial pressure; DAP: Diastolic arterial pressure; MAP: Mean arterial pressure; BR: Bradycardic response; TR: Tachycardic response; LV: Left ventricle; CWT: Circumferential wall tension.

## Competing interest

The authors declare that they have no competing interests.

## Authors’ contributions

EM: performed and coordinated the experiments; NEAL: performed the experiments; JFM: acquisition and analysis of data; CM: acquisition and analysis of data; KDA: data discussion and helped to draft the final manuscript; MCI: participated in the design, data discussion and helped to draft the manuscript; RBW: participated in design and coordination of this study; BR: participated in the data discussion and helped to draft the final manuscript; LBMM: conceived the study and participated in its design and coordination. All authors have read and approved the final manuscript.
